# Mammalian Base Excision Repair: Functional Partnership between PARP-1 and APE1 in AP-Site Repair

**DOI:** 10.1371/journal.pone.0124269

**Published:** 2015-05-28

**Authors:** Rajendra Prasad, Nadezhda Dyrkheeva, Jason Williams, Samuel H. Wilson

**Affiliations:** 1 Genome Integrity and Structural Biology Laboratory, NIEHS, National Institutes of Health, Research Triangle Park, North Carolina, United States of America; 2 Epigenetics and Stem Cell Biology Laboratory, NIEHS, National Institutes of Health, Research Triangle Park, North Carolina, United States of America; University of Massachusetts Medical School, UNITED STATES

## Abstract

The apurinic/apyrimidinic- (AP-) site in genomic DNA arises through spontaneous base loss and base removal by DNA glycosylases and is considered an abundant DNA lesion in mammalian cells. The base excision repair (BER) pathway repairs the AP-site lesion by excising and replacing the site with a normal nucleotide via template directed gap-filling DNA synthesis. The BER pathway is mediated by a specialized group of proteins, some of which can be found in multiprotein complexes in cultured mouse fibroblasts. Using a DNA polymerase (pol) β immunoaffinity-capture technique to isolate such a complex, we identified five tightly associated and abundant BER factors in the complex: PARP-1, XRCC1, DNA ligase III, PNKP, and Tdp1. AP endonuclease 1 (APE1), however, was not present. Nevertheless, the complex was capable of BER activity, since repair was initiated by PARP-1’s AP lyase strand incision activity. Addition of purified APE1 increased the BER activity of the pol β complex. Surprisingly, the pol β complex stimulated the strand incision activity of APE1. Our results suggested that PARP-1 was responsible for this effect, whereas other proteins in the complex had no effect on APE1 strand incision activity. Studies of purified PARP-1 and APE1 revealed that PARP-1 was able to stimulate APE1 strand incision activity. These results illustrate roles of PARP-1 in BER including a functional partnership with APE1.

## Introduction

Cellular DNA is constantly exposed to endogenous and exogenous genotoxic stressors, including environmental genotoxicants, irradiation and endogenous DNA damaging-agents [1–4]. These physical and chemical agents result in AP-sites and other lesions in DNA. AP-sites are among the most common DNA lesions, and it has been estimated that under normal physiological conditions >10,000 AP-sites are produced in each cell per day in higher eukaryotes [5,6]. Overexposure to genotoxicants can induce even higher levels of AP-sites that can exceed the capacity of the DNA repair systems [7,8]. This can have adverse consequences since failure to repair AP-sites can disrupt DNA transactions and lead to cytotoxic strand breaks, mutations and genomic instability [4,9–11].

Although there are multiple and overlapping DNA repair pathways in eukaryotic cells, the major pathway for repairing AP-sites, strand breaks and single-base damage is the base excision repair (BER) pathway [1,2,4,12–15]. An accepted model for mammalian BER involves two sub-pathways that are differentiated by the number of nucleotides replaced in the excision patch and the enzymes involved [16–19]. These BER sub-pathways are termed short patch or “single-nucleotide BER” (SN BER) and “long-patch BER” (LP BER). Repair is initiated after strand breaks, spontaneous base loss or removal by a DNA glycosylase [1,20,21]. The latter process results in the AP-site in DNA or the incised AP-site, depending on the DNA glycosylase involved. In the case of the intact AP-site, strand incision by AP endonuclease-1 (APE1) produces a single-nucleotide gap in DNA with 5′-deoxyribose phosphate (dRP) and 3′-hydroxyl groups at the margins [22,23]. This repair intermediate is processed by the bi-functional enzyme pol β that catalyzes 5′-dRP removal along with gap-filling DNA synthesis [24–28]. In the case of the LP BER sub-pathway, two or more nucleotides in the lesion-containing strand are replaced, either in a proliferating cell nuclear antigen-independent fashion by pol β and flap endonuclease 1 or in a proliferating cell nuclear antigen-dependent fashion by replicative polymerases and co-factors [16–19,29–32]. The final repair intermediate containing a nick is sealed by DNA ligase I or the complex of DNA ligase III and X-ray cross-complementing factor 1 (XRCC1) [33–35].

Through genetic and biochemical studies in many experimental systems, it is clear that base lesions and strand breaks can be rapidly repaired in cells and that multiple enzymes and scaffold factors interact to execute the repair processes [33,35–42]. In many cases, a macromolecular complex assembles at the site of a DNA lesion and the individual components of the complex coordinate the repair process [43–45]. Assembly of repair complexes is required for efficient repair. This strategy involving multiple interacting factors allows for a range of regulatory possibilities, first through post-translational modifications that influence repair complex stability and second through expression control of required components. In the case of DNA nicks and base lesions in mammalian cells, the precise interactions controlling repair at the site of a lesion are under investigation [42,46,47].

In addition to assembly of BER factors at DNA lesion sites, the factors are constitutively expressed in mammalian cells, and DNA-free macromolecular complexes of BER factors have been isolated using various biochemical techniques [48,49]. In a recent example, we used immunoaffinity-tagged pol β to isolate a multiprotein complex containing BER factors [44]. This pol β complex contained abundant poly(ADP-ribose) polymerase-1 (PARP-1) plus two BER enzymes, polynucleotide kinase/phosphatase (PNKP) and tyrosyl-DNA phosphodiesterase 1 (Tdp1), required in trimming the excision gap with a block at the 3′-hydroxyl group. Interestingly, the pol β complex did not include the primary AP-site strand incision enzyme APE1. Here, to understand implications of these results, we first investigated *in vitro* AP-site BER mediated by the pol β complex. The strand incision step in this case was provided by the PARP-1 AP lyase activity [50], and PNKP removed the O3′-phosphate blocking group produced by the β,δ-elimination of the PARP-1 lyase. Next, in studies of APE1-initiated AP-site BER by the pol β complex, our results suggested that PARP-1 was able to increase APE1 strand incision activity and conditions for this functional partnership were investigated with purified PARP-1 and APE1.

## Materials and Methods

### Materials

Synthetic oligodeoxyribonucleotides were from Oligos Etc., Inc. (Wilsonville, OR) and The Midland Certified Reagent Co. (Midland, TX). [α-^32^P]dCTP and [γ-^32^P]ATP (7000 Ci/mmol) were from Biomedicals (Irvine, CA). Optikinase was from USB Fermentas Inc. (Hanover, MD). Protease inhibitors complete (EDTA-free) were from Roche Molecular Diagnostics Corp. (Indianapolis, IN). Leupeptin, aprotinin, and phenylmethylsulfonyl fluoride were from Calbiochem (La Jolla, CA). Recombinant human APE1, pol β, DNA ligase I, uracil DNA glycosylase (UDG) with 84 amino acids deleted from the amino-terminus, XRCC1, and PARP-1 were purified as described previously [21,35,44,51–53]. *Escherichia coli* endonuclease III (Endo III) and *Escherichia coli* formamidopyrimidine N-glycosylase (Fpg) were from New England BioLabs (Ipswich, MA). PNKP and Tdp1 were generous gifts from Dr. Michael Weinfeld, Department of Experimental Oncology, Cross Cancer Institute, Edmonton, Alberta, Canada, and Yves Pommier, Laboratory of Molecular Pharmacology, NIH, Bethesda, USA, respectively. All purified proteins were diluted in a buffer that contained 50 mM HEPES, pH 7.5, 150 mM NaCl, 0.5 mM EDTA, 2 mM DTT, 100 μg/ml bovine serum albumin and 20% glycerol. Enzyme concentrations were measured by the Bio-Rad protein assay. Antibody to PARP-1 was from BD Biosciences PharMingen (San Jose, CA). Antibody to APE1 was described previously [35].

### 5′-end labeling of DNA for the APE1 incision assay

Dephosphorylated 34-mer oligodeoxyribonucleotide (5′-CTGCAGCTGATGCGC UGTACGGATCCCCGGGTAC-3′) or (5′-CTGCAGCTGATGCGC THFGTACGGATCCCCGGGTAC-3′) containing a uracil or the tetrahydrofuran (THF) analogue of the AP-site at position 16 was 5′-end labeled with Optikinase and [γ-^32^P]ATP. The 34-mer (5′-GTACCCGGGGATCCGTACGGCGCATCAGCTGCAG-3′) template was annealed with ^32^P-labeled oligonucleotides by heating the solution at 95°C for 3 min and then allowing it to slowly cool to 25°C. Unincorporated [γ-^32^P]ATP was removed using a MicroSpin G-25 column from GE HealthCare (Piscataway, NJ) according to the manufacturer’s suggested protocol.

### Preparation of AP-site DNA substrates

Two types of labeled oligonucleotide substrate were use. The ^32^P-labeled duplex oligonucleotide containing uracil was treated with UDG to generate the natural AP-site in intact DNA; however, in the case of duplex oligonucleotide containing the THF analogue, UDG treatment was omitted. Typically, 400 nM DNA substrate was pretreated in a reaction mixture containing 50 mM HEPES, pH 7.5, 0.5 mM EDTA, 2 mM dithiothreitol (DTT), 20 mM KCl, and 40 nM UDG. The reaction mixture was incubated for 30 min at 37°C. The 5′-end 6-FAM labeled DNA containing the AP-site analogue (5′-CATGGGCGGCATGAACTHFGGAGGCCCATCCTCACC-3′) was annealed to its appropriate complementary strand, as above, and this DNA substrate was used for APE1 kinetic studies.

### APE1 strand incision assay

For assay of strand incision activity by APE1, the reaction mixture contained 50 mM HEPES, pH 7.5, 20 mM KCl, 10 mM MgCl_2_, 0.5 mM EDTA, 2 mM DTT, 50 nM ^32^P-labeled DNA, 0.1 nM APE1, the indicated amounts of purified PARP-1, other purified BER factors, or the pol β complex. After incubation at 37°C for the periods specified, the reaction was terminated with an equal volume of DNA gel-loading buffer (2 M urea, 100 mM EDTA, 0.02% bromophenol blue, and 0.02% xylene cyanol). The reaction mixtures were then heated at 75°C for 2 min, and DNA substrates and products were separated by electrophoresis in a 16% polyacrylamide gel containing 8 M urea in 89 mM Tris-HCl, 89 mM boric acid and 2 mM EDTA, pH 8.8. A Typhoon PhosphorImager was used for gel scanning and imaging, and the substrate and product bands were quantified using ImageQuant Software.

### Kinetic measurements of APE1 strand incision activity

For kinetic measurements of the APE1 strand incision steady-state rate, the 5′-end 6-FAM labeled DNA substrate containing an AP-site (THF) was used. The reaction mixture contained 50 mM HEPES, pH 7.5, 20 mM KCl, 10 mM MgCl_2_, 0.5 mM EDTA, 0.1 mg/ml bovine serum albumin, and 100 nM DNA substrate. The reaction mixtures containing 25 to 500 nM PARP-1 were preincubated at room temperature for 5 min, as indicated in the figure legend. Then 0.5 nM APE-1 was added and incubated at 37°C. Time points were taken from 10 s to 5 min. Reactions were terminated with an equal volume of DNA gel-loading buffer, as above. After separation on a denaturing polyacrylamide gel, as above, the data were analyzed with ImageQuant software. Data were fit to an exponential equation to determine the steady-state rate of APE1 strand incision in the absence and presence of PARP-1; the values shown correspond to the 10 s time point.

### *In vitro* BER assay

The BER assay was performed, as described previously [54]. Briefly, the reaction mixture contained 50 mM HEPES, pH 7.5, 20 mM KCl, 5 mM MgCl_2_, 0.5 mM EDTA, 2 mM DTT, 4 mM ATP, 5 mM phosphocreatine, 100 μg/ml phosphocreatine kinase, 0.5 mM NAD^+^, 2.3 μM [α-^32^P]dCTP (specific activity, 1x10^6^ dpm/pmol), and 250 nM 35-mer double strand DNA with a uracil residue at position 15. The DNA was pre-treated with UDG. The BER reactions were then initiated either by addition of the pol β complex, as indicated in figure legends, or purified BER factors. The final reaction mixture concentrations of the purified factors were as follows: PARP-1 (200 nM), XRCC1 (200 nM), PNKP (150 nM), DNA ligase I (250 nM), pol β (20 nM); some BER reaction mixtures also contained Tdp1 (100 nM). The incubation was at 37°C. APE1 was omitted in the case of studies of APE1-independent BER; however, for BER with APE1, the reaction mixture contained 0.1 nM APE1. Aliquots were withdrawn from the reaction mixture at the indicated intervals, and the reaction was terminated with an equal volume of DNA gel-loading buffer. Measurements of reaction products and data analyses were performed as above.

### Characterization of PARP-1 cleavage products

For characterization of PARP-1 cleavage products, the reaction mixture containing 50 mM HEPES, pH 7.5, 20 mM KCl, 0.5 mM EDTA, 2 mM DTT, 10 mM MgCl_2_ and 200 nM ^32^P-labeled AP-site DNA was assembled on ice. Then the reaction mixture was supplemented either with 200 nM PARP-1, 4 U of Endo III, 5 U Fpg, 10 nM APE1, 100 nM PNK, or 0.1 M NaOH, as indicated in the figure legend. Note that MgCl_2_ was omitted in the reaction mixture with PARP-1. Reaction mixtures were incubated at 37°C for 15 min. After incubation, the reactions were terminated with an equal volume of DNA gel-loading buffer, as above; however, in the case of the NaOH treated sample, NaOH was neutralized with 0.1 N HCl before addition of DNA gel-loading buffer. The reaction mixtures were then heated at 75°C for 2 min, and denaturing polyacrylamide gel electrophoresis was employed to separate DNA substrates and products. A Typhoon PhosphorImager was used for gel scanning and imaging.

### Quantification of the major BER factors in the pol β complex

Immunoaffinity-tagged pol β was used to isolate a multiprotein complex containing BER factors from mouse embryonic fibroblast (MEF) cells, and the composition of the complex was determined by mass spectrometry [44]. The Spectrum Mill software suite from Agilent was used to analyze the mass spectrometry data and the relative amounts of these factors in the complex were estimated using the Total Protein Spectral Intensity as an approximation of protein abundance similar to the previously described method [55]. The pol β complex contained abundance of pol β along with PARP-1, DNA ligase III, XRCC1, PNKP and Tdp1. The amounts of these factors in the complex relative to pol β were determined and presented in a histogram (S1 Fig).

### Immunoblotting with extracts

Extracts were prepared from wild-type MEF cells in the log phase of growth, as described [56]. Portions, along with purified PARP-1 and APE1 used as standards, were separated by Nu-PAGE 4–12% Bis-Tris mini-gel and transferred onto nitrocellulose membrane. The membrane was incubated with 5% nonfat dry milk in Tris-buffered saline containing 0.1% (v/v) Tween 20 (TBS-T) and probed with antibody to PARP-1. Goat anti-mouse IgG conjugated to horseradish peroxidase (1:10,000 dilution) was used as secondary antibody, and the immobilized horseradish peroxidase activity was detected by enhanced chemiluminescence (ECL). The membrane was stripped by incubation in Restore Western Blot Stripping Buffer (Thermo Scientific, Rockford, IL) for 30 min at room temperature, followed by two washes with TBS-T. Then, the membrane was probed with antibody to APE1. Data were analyzed using ImageQuant 400 (GE Healthcare, Piscataway, NJ). APE1 and PARP-1 protein quantifications were obtained by comparing the signals with the extract and protein standards over linear ranges of proportionality for each (S1 Table).

## Results

### APE1-independent BER by the pol β complex

**Fig 1:**
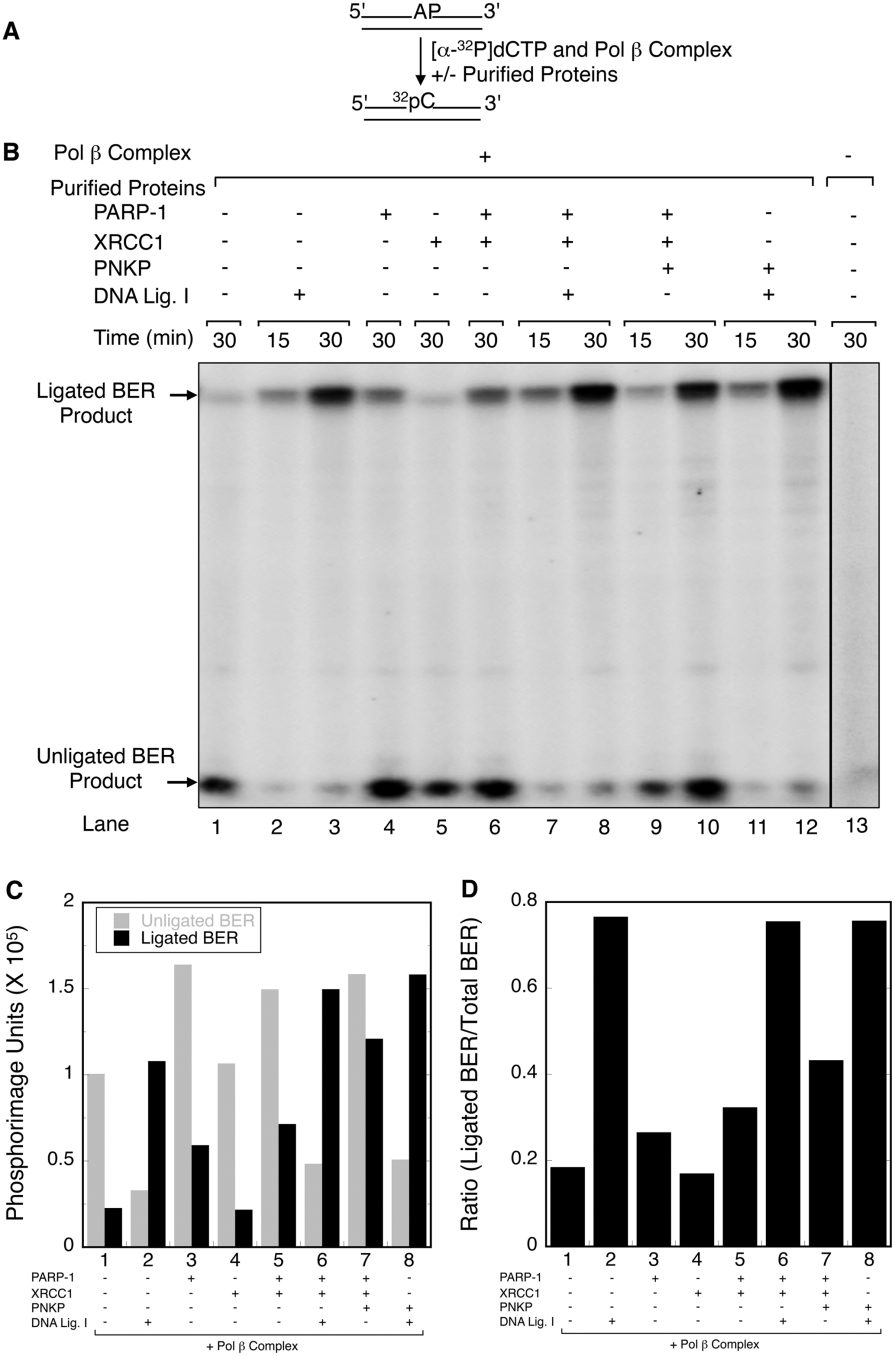
Effect of purified BER factors on APE1-independent BER by the pol β complex. (A) A schematic representation of the DNA substrate containing the AP-site and the reaction scheme is shown. (B) BER activity of the pol β complex was evaluated on an AP- site-containing DNA substrate by measuring incorporation of [α-^32^P]dCMP as a function of different components in the reaction mixture and incubation time. Reaction conditions and product analysis are described under Materials and Methods. AP-site DNA was incubated with the pol β complex in the presence (+) or absence (-) of purified BER factors including PARP-1 XRCC1, PNKP, DNA ligase I, as indicated at the top of the phosphorimage. Lane 13 represents the result after incubation of the reaction mixture without the pol β complex or purified proteins. Incubation was at 37°C for 15 and/or 30 min. The reaction products were separated by electrophoresis in a 16% polyacrylamide gel containing 8 M urea. A Typhoon PhosphorImager was used for gel scanning and imaging. The positions of the unligated BER product and ligated BER product are indicated. (C) AP-site DNA was incubated with the pol β complex in the presence (+) or absence (-) of purified BER factors, as indicated below the histogram. The ligated and unligated BER products at 30 min incubation were quantified using ImageQuant software and plotted in a histogram. The grey and black bars represent unligated and ligated BER products, respectively. (D) A histogram illustrating the ratios of ligated BER product to total BER products (both ligated plus unligated BER products) is shown.

The relative abundance of BER factors in the pol β complex was determined by mass spectrometry, and the results are shown in S1 Fig. Initially, we wished to measure the capacity of the pol β complex to conduct AP-site BER *in vitro*. BER reaction mixtures were assembled with AP-site containing duplex DNA either with the pol β complex alone or by supplementing the complex with purified BER factors (Fig 1 and S2 Fig). Repair was monitored by incorporation of [α-^32^P]dCMP replacing the AP-site. Quantification of the formation of both ligated and unligated BER products in the experiment in Fig 1 is shown in Fig 1C and 1D. The pol β complex alone was able to initiate AP-site repair as revealed by accumulation of the unligated BER intermediate (Fig 1B, lane 1). However, only a weak signal reflecting ligation of the intermediate was observed, in spite of the presence of DNA ligase III in the pol β complex. In light of the results in Fig 1B, lane 1, we examined the effect of supplementing the reaction mixtures with purified BER factors.

Addition of purified DNA ligase I resulted in formation of the ligated BER product (Fig 1B, lanes 2 and 3). Addition of purified PARP-1 produced an increase in both unligated and ligated BER products (Fig 1B, *c*.*f*., lanes 1 and 4), whereas addition of XRCC1 had little effect (Fig 1B, *c*.*f*., lanes 1 and 5; lanes 4 and 6). Addition of DNA ligase I to the PARP-1 and XRCC1 supplemented reaction mixtures produced a modest increase in BER products compared with addition of ligase I alone (Fig 1B and 1C, *c*.*f*., lanes 2–3 and 7–8). Interestingly, supplementing the pol β complex with PNKP increased the ligated BER product (Fig 1B, *c*.*f*., lanes 6 and 10), indicating that DNA ligase III in the complex was sufficient in this case to ligate the BER intermediate. Finally, addition of PNKP to the ligase I supplemented reaction mixture produced slightly more ligated product than addition of ligase I alone (Fig 1B, *c*.*f*., lanes 3 and 12, and Fig 1C). The ratios of ligated BER product to total BER products was higher in each case with the addition of DNA ligase I (Fig 1D). In a separate experiment with additional controls, similar results were obtained (S2 Fig).

To explain the AP-site BER in the absence of APE1 and the stimulation of BER by purified PARP-1 and PNKP, we examined the products of AP-site strand incision by PARP-1. Products of PARP-1 strand incision [50] on a natural AP-site substrate were measured and compared with those of three reference enzymes and NaOH treatment. The 5′-end ^32^P-labeled AP-site DNA was incubated either with purified PARP-1, APE1, Endo III, or Fpg or treated with NaOH (S3A Fig). Endo III cleaved the AP-site resulting in the doublet that is a signature of its β elimination AP lyase reaction; Fpg conducted β,δ-elimination AP lyase activity [57,58] and produced the 3′-phosphate containing product. NaOH treatment products served as a reference for β- and β,δ-elimination (S3A Fig, lane 6). APE1 yielded the 3′ OH-containing product, that migrated slower than the β,δ-elimination product of Fpg and NaOH treatment (S3A Fig, lane 2). PARP-1 mainly produced β-elimination products (i.e., a doublet as seen for Endo III), along with a weak band corresponding to the 3´-phosphate containing product of Fpg, *i*.*e*., the β,δ-elimination product (S3A Fig, lane 3). In a separate experiment to confirm the identity of this weak band, incubation of the substrate with both PARP-1 and PNKP resulted in formation of the 3′ OH-containing product (S3B Fig, *c*.*f*., lanes 1 and 2) with only minimal residual β,δ-elimination 3′-phosphate product remaining.

These results on PARP-1 strand incision products enabled us to rationalize the APE1-independent BER activity of the pol β complex: the PARP-1 AP lyase activity initiated AP-site BER by producing strand incision and the β,δ-elimination product with a O3´-phosphate block; the phosphate blocking group was removed by PNKP, generating the substrate for pol β gap-filling. Nevertheless, we suspected this APE1-independent BER activity of the complex was relatively weak, especially in light of the minimal production of the β,δ-elimination product. Thus, the effect of adding purified APE1 to the BER reaction mixture with the pol β complex was determined (S4 Fig). APE1 produced a strong stimulation of BER activity by the pol β complex, as expected. This suggested that the BER activity in the absence of APE1 might represent a backup system in the event of APE1 deficiency [59,60].

To further examine the results in Fig 1, S2 and S3 Figs indicating that PARP-1 can incise the AP-site substrate, we conduced *in vitro* BER assays with a mixture of purified proteins under similar reaction condition as in Fig 1. The results failed to reveal significant repair activity when the proteins where added to the reaction mixture individually (S5 Fig, lanes 1–7), although a weak near-background level of dCMP incorporation was observed with pol β (S5 Fig, lane 6). In the absence of DNA ligase I, formation of the unligated BER intermediate depended on pol β, but did not depend on Tdp1 (S5 Fig, lanes 11 and 13). Formation of the ligated BER product with the complete mixture of purified BER proteins depended on pol β, as expected (S5 Fig, lanes 14 and 15). The unligated BER intermediate was observed in the reaction mixtures with PARP-1, XRCC1, PNKP and pol β (S5 Fig, lanes 11 and 13), and the unligated BER intermediate was converted to ligated BER product upon addition of DNA ligase I (S5 Fig, lane 15). In summary, the results described so far revealed that the pol β complex was able to support AP-site BER in the absence of APE1, albeit at a relatively low level.

### Effect of PARP-1 on APE1-dependent BER

**Fig 2:**
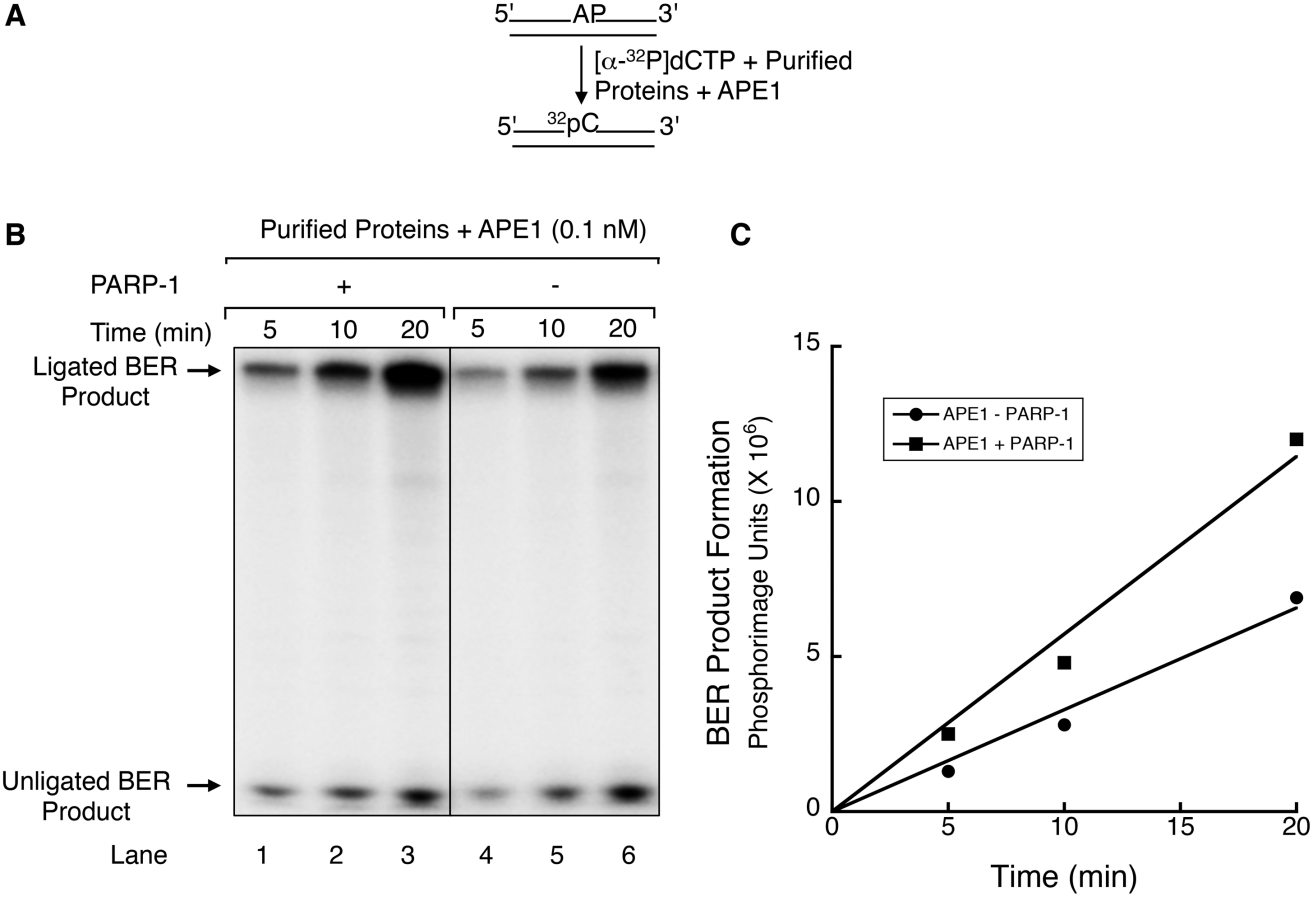
Effect of PARP-1 on APE1-dependent BER. (A) A schematic representation of the DNA substrate containing the AP-site and the reaction scheme is shown. The BER reaction conditions and product analysis are described under Materials and Methods. (B) The BER reaction mixtures containing purified proteins XRCC1, PNKP, DNA ligase I and APE1 were supplemented either with PARP-1 (lanes 1–3) or dilution buffer (lanes 4–6). Repair was initiated by transferring the reaction mixtures to 37°C. Aliquots were withdrawn at 5, 10 and 20 min. The reaction products were analyzed as in Fig 1. The positions of the BER intermediate (unligated) and ligated BER products are indicated. (C) Quantification of the BER products was performed using ImageQuant software and data plotted as a function of incubation time (min). The plot demonstrates that BER product formation was linear during the 20 min incubation and that PARP-1 stimulated BER at least 2-fold as compared to the reaction without additional PARP-1.

As noted above, addition of purified APE1 strongly increased the pol β complex BER activity (S4 Fig) and a similar effect of APE1 was obtained with the purified BER protein mixture. Since PARP-1 is an abundant nuclear protein and exceeds the concentration of APE1 (S1 Table), PARP-1 probably binds to and occupies the AP-site immediately upon its generation [44,50,61]. Evidently, APE1 is able to gain access to its AP-site substrate in the presence of bound PARP-1. To test this idea, we first examined the effect of PARP-1 on BER by the mixture of purified BER proteins in the presence of a limiting concentration of APE1 (Fig 2). Interestingly, BER activity was higher in the presence than in the absence of PARP-1. In additional experiments, we found the stimulatory effect of PARP-1 on BER was not dependent on PARP-1’s auto-poly(ADP-ribosyl)ation (S6 Fig). Since APE1 was limiting in these experiments, the results are consistent with PARP-1 stimulation of APE1, and this possibility was explored in the experiments to be described below.

### Increase in APE1 strand incision activity by PARP-1

**Fig 3:**
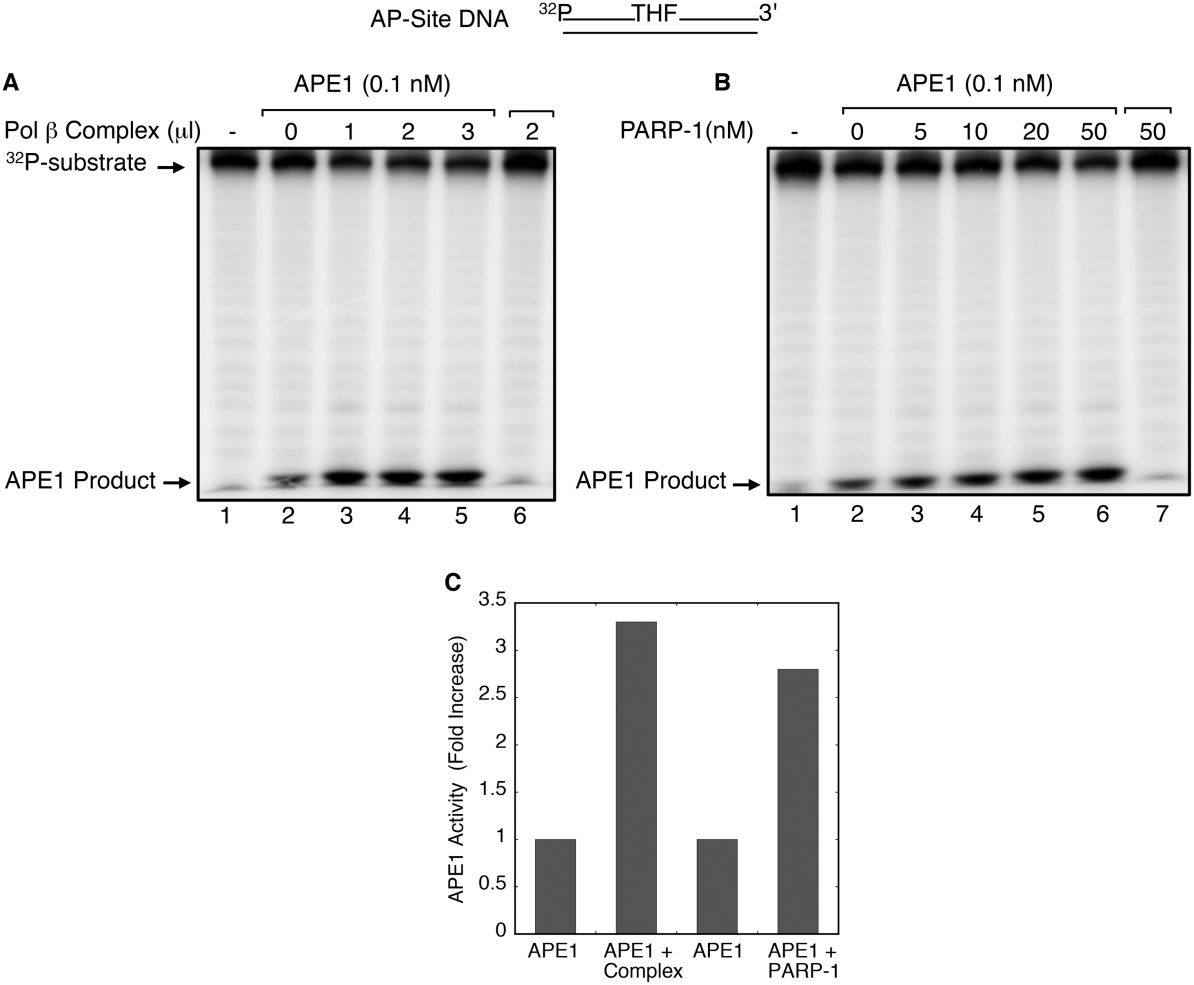
Stimulation of APE1 activity by the pol β complex or purified PARP-1. A schematic representation of the DNA substrate containing the AP-site analogue THF is shown at the top. The reaction conditions and product analysis are described under Materials and Methods. (A) APE1 incision reactions were assembled on ice either with increasing amounts of pol β complex (A) or with increasing amounts of purified PARP-1 (B) The incision reaction was initiated by addition of 0.1 nM APE1 and transferring the reaction mixtures to 37°C for 10 min. The reaction products were analyzed as in Fig 1. The positions of the ^32^P-labeled substrate and the product of APE1 strand incision are indicated. A representative phosphorimage of two repeats is illustrated. (C) Quantification of the APE1 product formed at the highest amount of pol β complex (3 μl) and the highest concentration of PARP-1 (50 nM) reveal an approximately 3-fold increase in APE1 activity as compared to that of APE1 alone. The mean of two repeats is illustrated.

Since the BER activity of the pol β complex was much higher in the presence of purified APE1, we were curious to test whether the complex could partner with APE-1 and modulate the APE1 strand incision activity. Thus, we examined APE1 strand incision activity directly with increasing concentrations of the pol β complex (Fig 3A) or with purified PARP-1 alone (Fig 3B). A 5′-end ^32^P-labeled DNA substrate containing the AP-site analogue THF was incubated with APE1 alone or with APE1 and increasing amounts of pol β complex or purified PARP-1. APE1 product formation increased with increasing amounts of pol β complex (Fig 3A, lanes 3–5), corresponding to a 3-fold increase with the highest amount of complex (Fig 3C). A similar effect on APE1 strand incision was observed in reaction mixtures with increasing concentrations of purified PARP-1 (Fig 3B and 3C). In control incubations, strand incision activity in the absence of APE1 was negligible. In additional experiments, a similar effect on APE1 strand incision was found with a substrate containing the natural AP-site (S7 Fig). In contrast, two other purified BER factors found in the pol β complex, XRCC1 and PNKP, failed to produce a stimulatory effect on APE1 activity (S8A and S8B Fig). Further, the PARP-1 stimulation of APE1 was not changed by the presence of the PARylation substrate NAD^+^ (S9 Fig). Overall, these results are consistent with the idea that the stimulatory effect of PARP-1 on APE1-dependent BER activity (Fig 2) was due to an increase in APE1 strand incision. The stimulatory effect in these experiments was not due to the weak AP lyase activity of PARP-1.

### Kinetic studies of the effect of PARP-1 on APE1 strand incision activity

**Fig 4:**
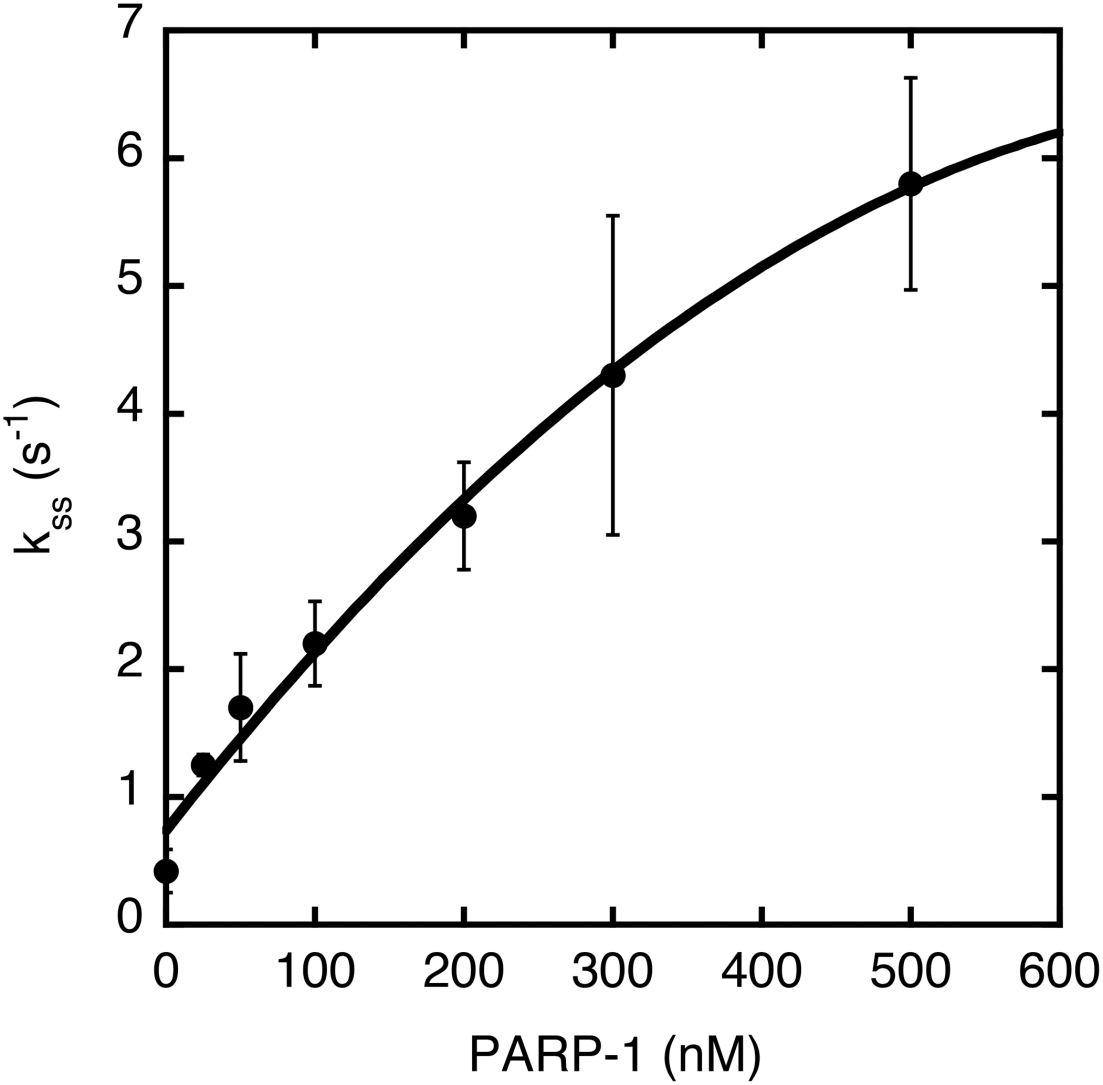
Effect of PARP-1 on the steady-state rate of AP-site incision catalyzed by APE1. The DNA substrate with THF (100 nM) was preincubated with 25–500 nM PARP-1. After adding 0.5 nM APE1, the reaction mixture was incubated for 10 s to 5 min at 37°C. The reaction conditions and data analysis are described in Materials and Methods. The data representing the reaction products were fitted to an exponential equation to determine the steady-state rate of the APE1 incision reaction in the absence and presence of PARP-1. The average from three repeats is represented.

We next performed quantitative kinetic studies of APE1 strand incision activity using an APE1 substrate containing the AP-site analogue THF. Steady-state conditions were chosen, and the steady-state rate constant of APE1 alone (~0.5 s^-1^) was found to be similar to that reported previously [62–64]. Next, various concentrations of purified PARP-1 were preincubated with the DNA substrate prior to addition of APE1. AP-site incision was measured at different time points, and the data were fit to an exponential equation to determine the steady-state rate of APE1 incision in the presence of PARP-1. The rate of APE1 strand incision was >10-fold higher at the highest concentration of PARP-1 tested (Fig 4).

## Discussion

The results described here point to a PARP-1 stimulatory effect on APE1-dependent BER *in vitro*. PARP-1 and APE1 appear to have a functional interaction in BER since PARP-1 can stimulate the strand incision activity of APE1. Among other features, questions about APE1 and PARP-1 transactions at the AP-site during BER are not clear, but are interesting because both APE1 and PARP-1 are known to recognize the AP-site in double-strand DNA, and both enzymes can bind to the same BER intermediate after APE1 incision at the AP-site [39,50,65]. For example, in kinetic studies, APE1 has high affinity for the substrate AP-site, as well as for the APE1 reaction product, the incised AP-site [52]. This feature enables APE1 to remain bound after rapid incision of the AP-site, and this could influence the efficiency of overall BER pathway by promoting substrate channeling between the APE-1’s product that is the pol β substrate.

PARP-1 is considered to be an abundant nuclear protein, and we were interested in comparing the APE1 level in mouse fibroblasts with the level of PARP-1. As shown in S1 Table, we found that PARP-1 was >10-fold more abundant than APE1. The excess of PARP-1 over APE1 may be even greater, in light of APE1’s nucleolus and cytoplasmic compartmentalization [66]. In addition, APE1 forms complexes with RNA and this could reduce the effective APE1 concentration available for activity at the PARP-1 bound AP-site [66,67]. The suggestion from these considerations is that PARP-1 may occupy the AP-site and interact with APE1 as it becomes available. Nevertheless, the molecular mechanism of the apparent dual occupancy of the AP-site DNA by PARP-1 and APE1 is unclear, especially because crystal structures of APE1 in complex with its product show intimate contact with the AP-site [68,69].

In the initial experiments here, we found that the pol β complex was able to support BER of AP-site DNA in the absence of APE1. This endogenous pol β complex BER activity depended on PARP-1 AP lyase strand incision and β,δ-elimination, plus PNKP unblocking of the O3′ group at the gap margin of the BER intermediate (Fig 1). The significance of the pol β complex BER activity could be that it functions in a BER sub-pathway that does not require APE1. Yet, this “endogenous” BER activity of the pol β complex was only weak compared to the AP-site BER activity in the presence of purified APE1 (S4 Fig); the weak endogenous activity was consistent with the low level of the PARP-1 AP lyase activity [50]

We examined the possibility of a functional partnership between the APE1 strand incision activity and the pol β complex. In assays for APE1 strand incision on a THF-containing substrate, the pol β complex stimulated APE1 activity (Fig 3). This stimulation of APE1 activity also was consistent with that observed with purified PARP-1 alone (Fig 3), and in kinetic analysis an increase in the steady-state rate constant of APE1 strand incision was observed as a function of increasing PARP-1 concentrations (Fig 4). With the observations of a functional interaction between PARP-1 and APE1 during the incision step at the AP-site, we tested the possibility of a stable complex between the two purified enzymes or of a ternary complex between DNA and the two enzymes. Electrophoretic mobility shift assays failed to reveal any complex formation between PARP-1 and APE1 (data not shown). Details of the interaction of APE1 at the PARP-1 bound AP-site remain to be clarified.

**Fig 5:**
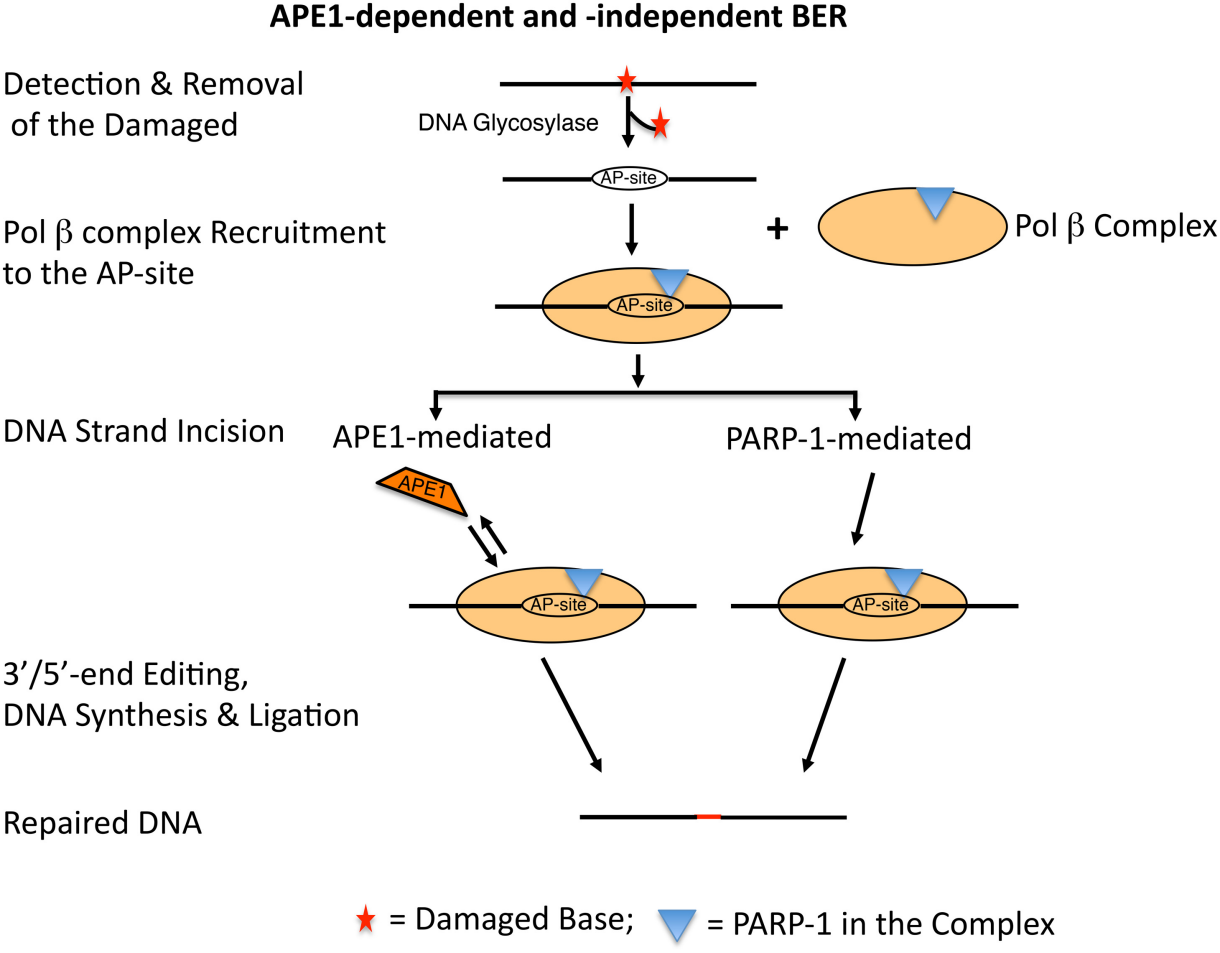
A model illustrating APE1-dependent and-independent mammalian BER coordinated by BER factors in the pol β complex. AP-site lesions in DNA that are formed by spontaneous hydrolysis of the *N*-glycosylic bond or by removal of inappropriate bases by DNA *N*-glycosylases are recognized by PARP-1 [44,50,61]. By virtue of the presence of PARP-1 in the pol β complex, the complex is recruited to the AP-site in DNA. Upon binding to AP-site, PARP-1 is auto-poly(ADP-ribosyl)ated [44]. While the complex remains bound to the AP-site DNA strand, BER may proceed either by an APE1-dependent (*left-hand* side of the scheme) or APE1-independent (*right-hand* side of the scheme) pathway. In the case of the APE1-dependent pathway, APE1 incises the AP-site, while the complex is still bound to the AP-site. The dRP removal, DNA synthesis and ligation steps are conducted. On the other hand, in situations where APE1 is deficient, APE1-independent BER operates where PNKP plays a central role [60]. In this case, for example, the complex bound at the AP-site incises the DNA strand by its PARP-1’s lyase activity [50]. Tdp1 and/or PNKP trim or edit the 3′blocked group to generate the 3′-OH necessary for the DNA synthesis and ligation steps, respectively. PAPR-1 is depicted as the blue triangle in the pol β complex.

Based on these observations, we propose a working model (Fig 5) where PARP-1 in the pol β complex recognizes and binds at the AP-site containing DNA strand. As illustrated in Fig 5, while the complex remains bound to the AP-site DNA strand, BER can occur either by an APE1-dependent (left-hand side of the scheme) or APE1-independent (right-hand side of the scheme) pathway. In the case of the APE1-dependent pathway, APE1 incises the AP-site, while the complex is still bound to the AP-site. The dRP removal, DNA synthesis and ligation steps are conducted by pol β and DNA ligase III/XRCC1 or DNA ligase I, respectively. However, in cells where APE1 is deficient or down regulated [59,60], the APE1-independent pathway may operate, albeit at a relatively low level. In this case, PARP-1 bound at the AP-site incises the DNA strand by its β- and β,δ-elimination activities [50]. Tdp1 and/or PNKP are required to trim or edit the 3´blocked group to generate the 3´-OH necessary for the DNA synthesis and ligation steps.

Numerous functional and/or physical partnerships among BER factors have been described. Well-known examples of functional partnerships include APE1 stimulation of the pol β dRP lyase activity, APE1 stimulation of the DNA glycosylases TDG and OGG1, XRCC1 stimulation of APE1 strand incision activity, XRCC1 stimulation of PNKP 3′-phosphatase activity, and APTX stimulation of OGG1 activity [41,70–73], among others. XRCC1 and PARP-1 appear to function as scaffold proteins for BER factors and have physical interactions between themselves and several other BER factors [74]. However, in the case of pol β and APE1, a physical interaction was observed only in the presence of a DNA substrate [41,70].

Macromolecular complexes containing BER factors [35,36,44,46,75,76], and the inter-protein interactions summarized here present opportunities for regulation of BER. One consideration is that a proximity effect of multiple enzymes in association with lesion-containing DNA presents a logical regulatory strategy for the cell [39]. Post-translational modifications and redox changes [77] that alter these protein-protein interactions also could provide a means of BER regulation, as is the case, for example, in regulation of the PNKP-PARP-1 interaction through PNKP phosphorylation [78] and the regulation of the XRCC1-PNKP interaction by phosphorylation of XRCC1 [79]. However, a functional partnership between factors need not involve a stable complex that can be readily observed experimentally. For example, the functional partnership observed here between PARP-1 and APE1 did not involve a stable complex of either the protein-protein type or the multi-protein-DNA complex type.

## Supporting Information

S1 Fig Apparent abundances of BER factors in the pol β complex. Representative experiment of seven replicates showing the estimated relative abundance of BER factors in the pol β complex. Abundances were estimated by summing the areas under the extracted ion chromatograms of the three most abundant ions attributed to each of these BER proteins similar to the method described by Silva, *et al*. [55] and also based upon the total protein spectral intensity value as calculated using the Spectrum Mill software from Agilent. The histogram illustrates the relative abundance of BER factors in the pol β complex.
(TIF)

S2 Fig Effect of purified BER factors on APE1-independent BER by the pol β complex. BER activity of the pol β complex was evaluated on an AP site-containing DNA substrate by measuring incorporation of [α-^32^P]dCMP as a function of supplementing with purified BER factors. Reaction conditions and product analysis are described under Materials and Methods. AP-site DNA (250 nM) was incubated in the presence of the pol β complex and with (+) or without (-) of purified BER factors, as indicated. The purified BER factors included PARP-1 (200 nM), XRCC1 (200 nM), PNKP (150 nM), Tdp1 (100 nM), and DNA ligase I (250 nM) alone or in various combinations, as indicated at the top of the phosphorimage. Lane 13 represents the reaction mixture without the pol β complex and purified proteins. Incubation was at 37°C for 40 min. The reaction products were analyzed as in Fig 1. The positions of the unligated BER product and ligated BER product are indicated.
(TIF)

S3 Fig Characterization of PARP-1 cleavage products. Reaction conditions and product analysis were as described under Materials and Methods. Schematic representation of ^32^P-labeled AP-site DNA is shown at the top of the phosphorimage. (A) The AP site-containing substrate was incubated with APE1 (5 nM, lane 2), PARP-1 (200 nM, lane 3), Endo III (4 units, lane 4), Fpg (5 units, lane 5), or NaOH (0.1 M, lane 6), and the reaction products were analyzed as described under Material and Methods. Lane 1 represents DNA alone. (B) In a separate experiment, the AP site-containing substrate was incubated either with PARP-1 (lane 1) or with both PARP-1 and PNKP (lane 2), as in (A). Incubation of the substrate with both PARP-1 and PNKP resulted in formation of the 3′ OH-containing product (compare lanes 1 and 2) with only minimal residual β,δ-elimination 3′-phosphate product remaining. The migration positions of the β-elimination (slower migrating doublet), β,δ-elimination (PO_4_) and APE1-incised (3′-OH) products are indicated.
(TIF)

S4 Fig Influence of APE1 on BER-mediated by the pol β complex. (A) A schematic representation of the DNA substrate containing the AP-site and the reaction scheme is shown. The reaction conditions and product analysis were as described under Materials and Methods. (B) Repair reactions without APE1 (lanes 1 and 2) or with APE1 (lanes 3 and 4) were initiated by the addition of the pol β complex. The incubation was at 37°C. Aliquots were withdrawn at 20 and 40 min in experiments without APE1, or 5 and 10 min with APE1, as indicated. The reaction products were analyzed as in Fig 1. The positions of the unligated BER product and ligated BER product are indicated.
(TIF)

S5 Fig Effect of purified BER factors on APE1-independent BER. A schematic representation of the DNA substrate containing the AP-site and the reaction scheme is illustrated at the top. The reaction conditions and product analysis are described under Materials and Methods. The repair reaction was assembled on ice with (+) or without (-) purified BER factors alone or in various combinations, as indicated at the top of the phosphorimage. Repair was initiated by transferring the reaction mixtures to 37°C, and incubation was for 40 min. The reaction products were analyzed as in Fig 1. The positions of the unligated BER product and ligated BER product are indicated.
(TIF)

S6 Fig Influence of NAD^+^ on the PARP-1-mediated stimulation of AP-site DNA BER with purified proteins. A schematic representation of the AP-site DNA substrate and the reaction scheme is illustrated at the top. The reaction conditions and product analysis are described under Materials and Methods. Repair reactions were supplemented either with (+) or without (-) 200 nM PARP-1, NAD^+^ (100 μM), or the PARP inhibitor 4-AN (100 μM), as indicated. The repair was initiated by transferring the reaction mixtures to 37°C. Aliquots were withdrawn at 2, 5, 10 and 20 min, as indicated. The reaction products were analyzed as in Fig 1. The positions of the unligated BER product and ligated BER product are indicated. In the reaction mixtures with the PARP-1 inhibitor 4-AN, PARylation was inhibited > 95%.
(TIF)

S7 Fig Stimulation of APE1 activity by the pol β complex or purified PARP-1. A schematic representation of the DNA substrate containing the natural AP site (AP) is shown at the top. The reaction conditions and product analysis are described under Materials and Methods. (A) APE1 incision reactions were assembled on ice either with increasing amounts of pol β complex (A) or with increasing amounts of purified PARP-1 (B). The incision reaction was initiated by addition of APE1 and transferring the reaction mixtures to 37°C for 10 min. The reaction products were analyzed as in Fig 1. The positions of the ^32^P-labeled substrate and product of APE1 strand incision are indicated. (C) Quantification of APE1 product formation at the highest amount of the pol β complex (3 μl) and the highest concentration of PARP-1 (50 nM) revealed an approximately 3-fold increase in APE1 activity as compared to that of APE1 alone.
(TIF)

S8 Fig Effect of PNKP or XRCC1 on APE1 activity. A schematic representation of the APE1 DNA substrate containing THF is illustrated at the top. The reaction conditions and product analysis are described under Materials and Methods. The APE1 incision reaction mixture was assembled on ice, either with increasing amounts PNKP (A), or XRCC1 (B), as indicated. The minus APE1 control lane is indicated. The incision reaction was initiated by adding APE1 and transferring the reaction mixtures to 37°C. After 10 min incubation, the reaction products were analyzed as in Fig 1. The positions of the ^32^P-labeled substrate and product of APE1 strand incision are indicated. Lane 1 in panel (A) represents substrate alone. The results of this analysis showed no increase in APE1 activity with addition of PNKP or XRCC1 as compared to that of APE1 alone.
(TIF)

S9 Fig Influence of NAD^+^ on the PARP-1-mediated stimulation of APE1 strand incision activity. A schematic representation of the DNA substrate containing THF is illustrated at the top. The reaction conditions and product analysis were as described under Materials and Methods and S7 Fig. The incision reaction mixture (10 μl) was assembled on ice with ^32^P-labeled AP-site DNA (50 nM) and increasing amounts of PARP-1 with (+) or without (-) NAD^+^, as indicated. Lanes 6 and 7 are minus APE1 controls. The incision reaction was initiated by addition of APE1 to the final concentration of 0.1 nM and transferring the reaction mixtures to 37°C. After 10 min incubation, the reaction products were analyzed as in Fig 1. The positions of the ^32^P-labeled substrate and product of APE1 strand incision are indicated. Quantification (not shown) of the APE1 products demonstrated an approximately 3-fold increase in APE1 strand incision activity as compared to that of APE1 alone, whereas the activity was not influenced by NAD^+^.
(TIF)

S1 Table Levels of APE1 and PARP-1 in MEF cells. Values of APE1 and PARP-1 in cell extracts were measured by quantitative immunoblotting as described under Materials and Methods using purified APE1 and PARP-1 as standards.
(TIF)
