# Supplementary figures and images for: Mammalian Base Excision Repair: Functional Partnership between PARP-1 and APE1 in AP-Site Repair
Source: PLoS One. 2015 May 28;10(5):e0124269. doi: 10.1371/journal.pone.0124269 (PMC4447435; doi:10.1371/journal.pone.0124269)

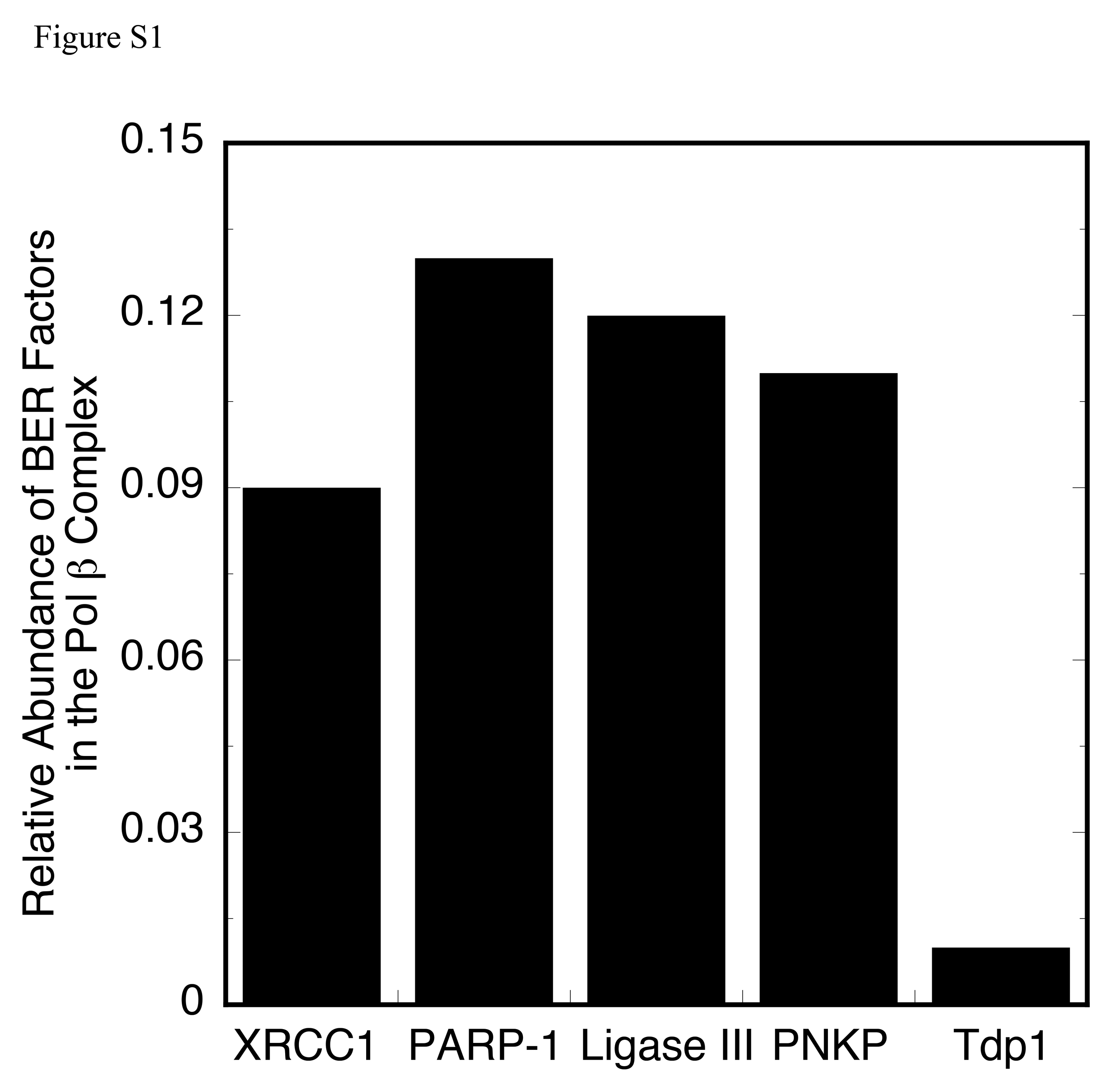

Supplement: S1 Fig — Representative experiment of seven replicates showing the estimated relative abundance of BER factors in the pol β complex. Abundances were estimated by summing the areas under the extracted ion chromatograms of the three most abundant ions attributed to each of these BER proteins similar to the method described by Silva, et al. [55] and also based upon the total protein spectral intensity value as calculated using the Spectrum Mill software from Agilent. The histogram illustrates the relative abundance of BER factors in the pol β complex. (TIF) [file pone.0124269.s001.tif]

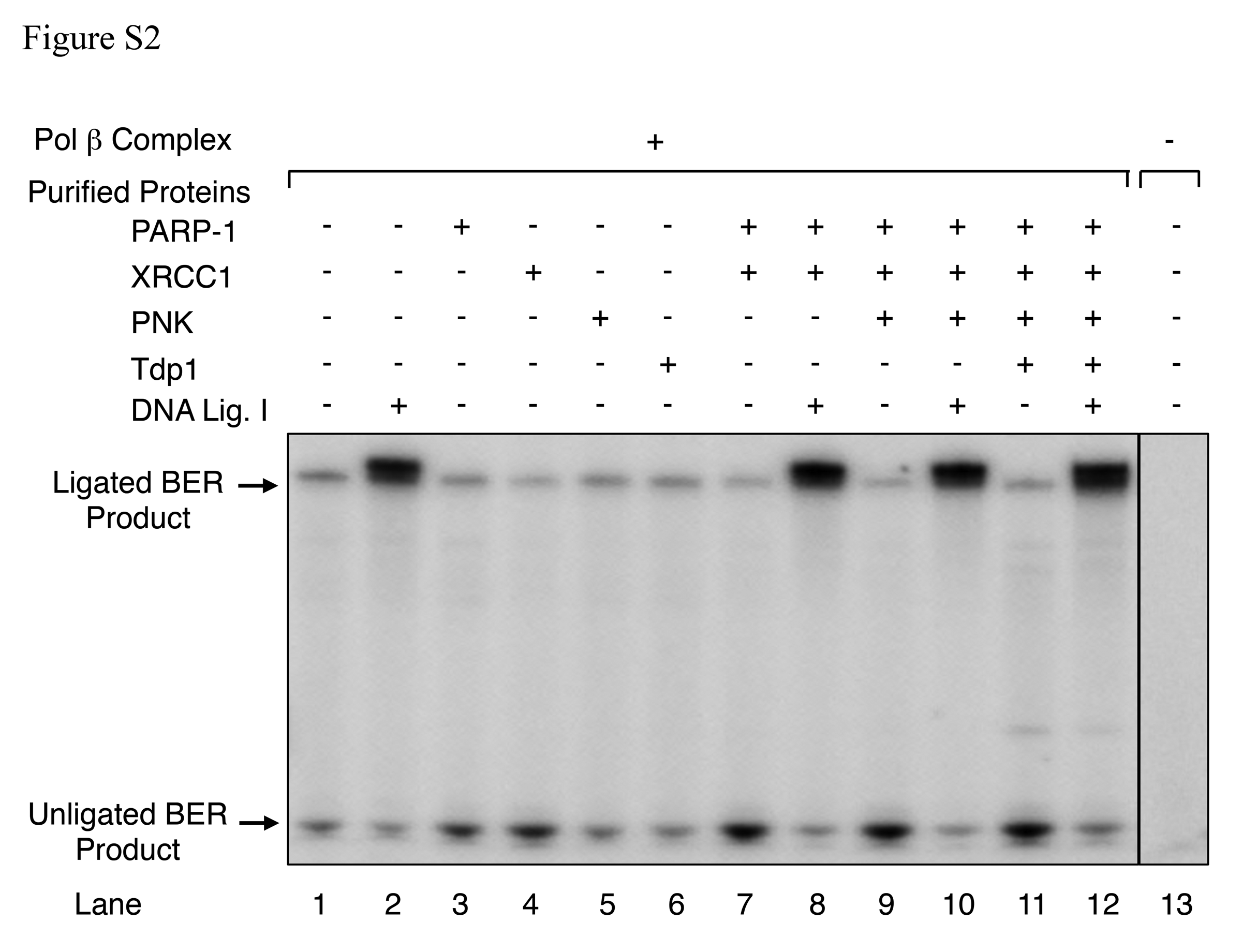

Supplement: S2 Fig — BER activity of the pol β complex was evaluated on an AP site-containing DNA substrate by measuring incorporation of [α-32P]dCMP as a function of supplementing with purified BER factors. Reaction conditions and product analysis are described under Materials and Methods. AP-site DNA (250 nM) was incubated in the presence of the pol β complex and with (+) or without (-) of purified BER factors, as indicated. The purified BER factors included PARP-1 (200 nM), XRCC1 (200 nM), PNKP (150 nM), Tdp1 (100 nM), and DNA ligase I (250 nM) alone or in various combinations, as indicated at the top of the phosphorimage. Lane 13 represents the reaction mixture without the pol β complex and purified proteins. Incubation was at 37°C for 40 min. The reaction products were analyzed as in Fig 1. The positions of the unligated BER product and ligated BER product are indicated. (TIF) [file pone.0124269.s002.tif]

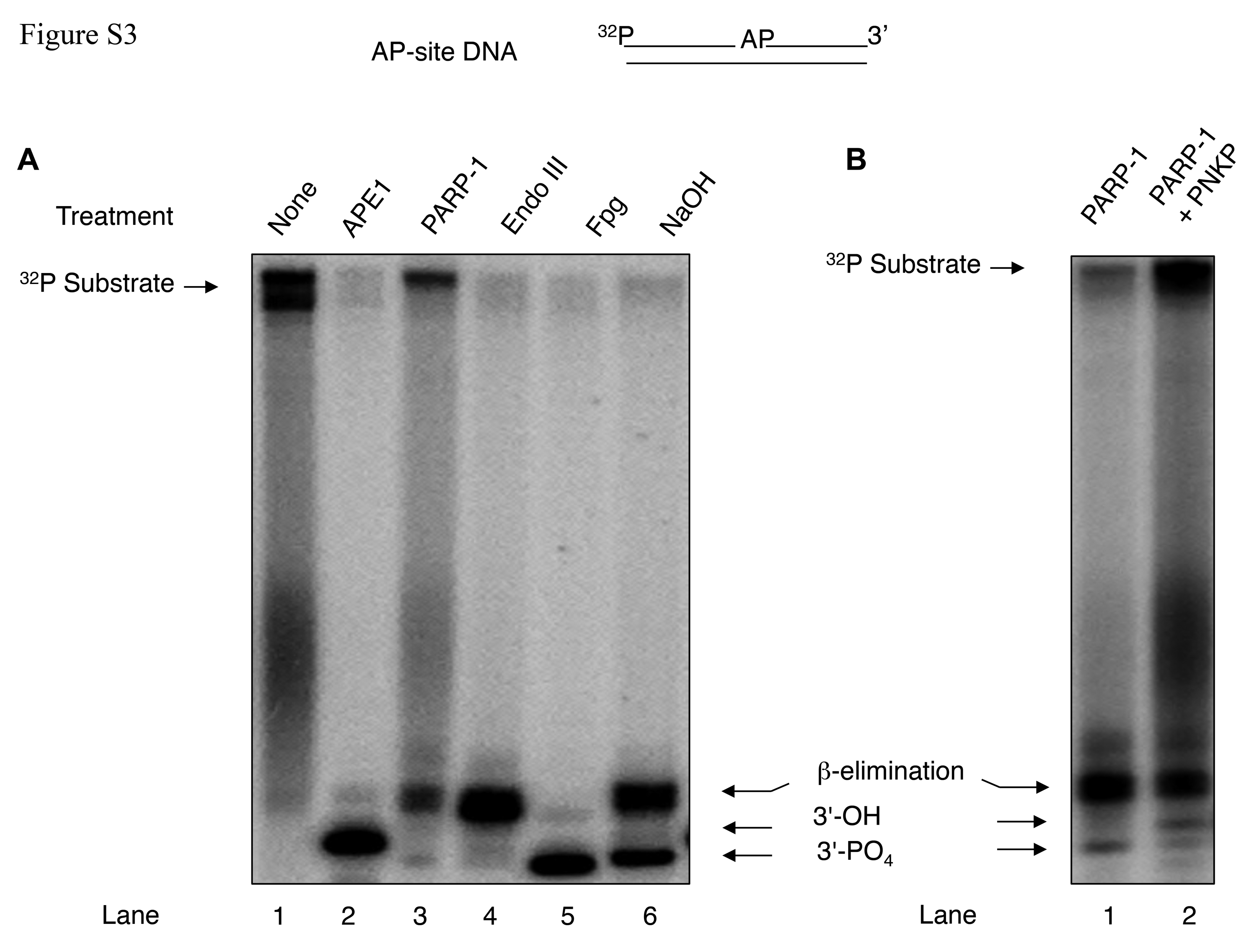

Supplement: S3 Fig — Reaction conditions and product analysis were as described under Materials and Methods. Schematic representation of 32P-labeled AP-site DNA is shown at the top of the phosphorimage. (A) The AP site-containing substrate was incubated with APE1 (5 nM, lane 2), PARP-1 (200 nM, lane 3), Endo III (4 units, lane 4), Fpg (5 units, lane 5), or NaOH (0.1 M, lane 6), and the reaction products were analyzed as described under Material and Methods. Lane 1 represents DNA alone. (B) In a separate experiment, the AP site-containing substrate was incubated either with PARP-1 (lane 1) or with both PARP-1 and PNKP (lane 2), as in (A). Incubation of the substrate with both PARP-1 and PNKP resulted in formation of the 3′ OH-containing product (compare lanes 1 and 2) with only minimal residual β,δ-elimination 3′-phosphate product remaining. The migration positions of the β-elimination (slower migrating doublet), β,δ-elimination (PO4) and APE1-incised (3′-OH) products are indicated. (TIF) [file pone.0124269.s003.tif]

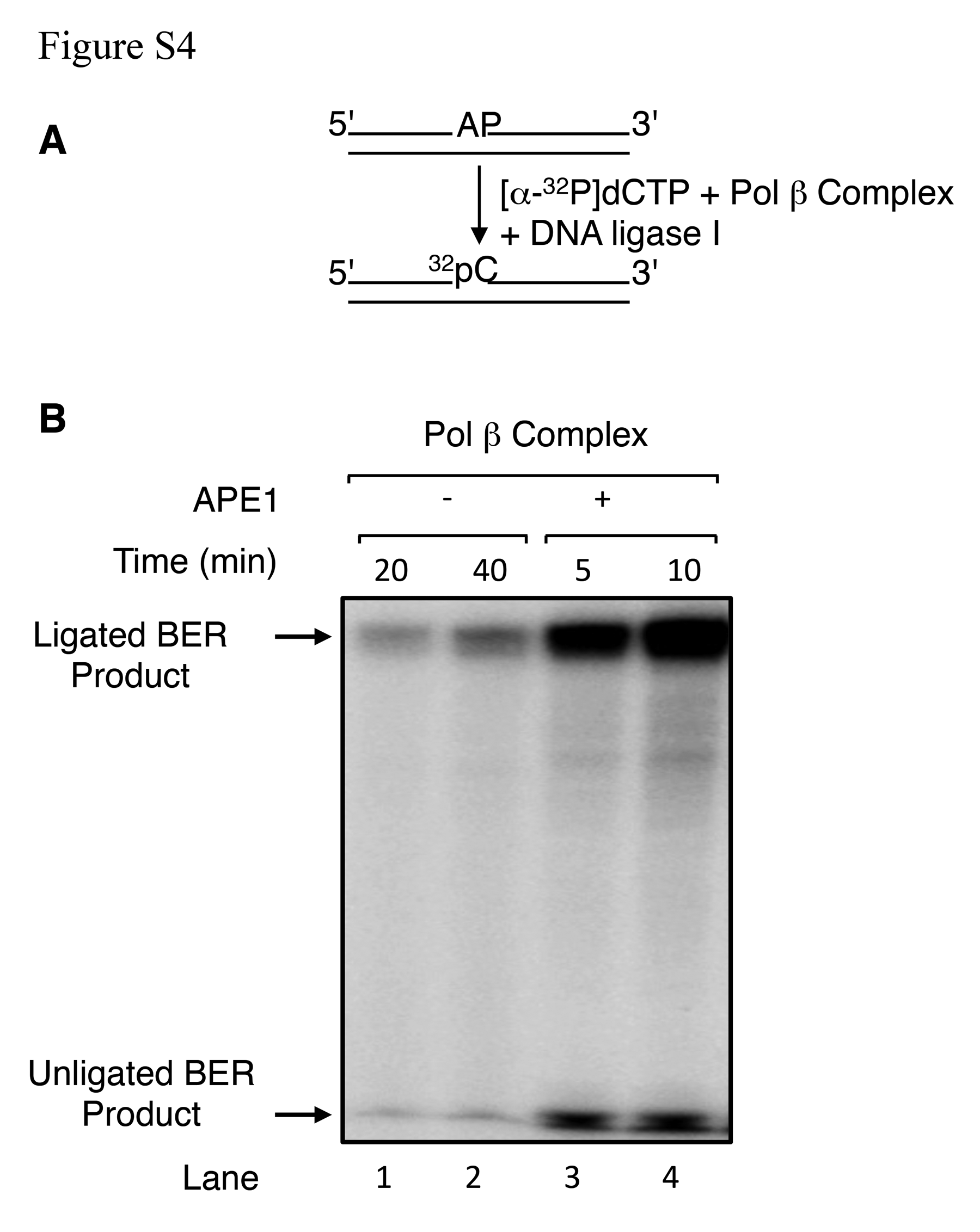

Supplement: S4 Fig — (A) A schematic representation of the DNA substrate containing the AP-site and the reaction scheme is shown. The reaction conditions and product analysis were as described under Materials and Methods. (B) Repair reactions without APE1 (lanes 1 and 2) or with APE1 (lanes 3 and 4) were initiated by the addition of the pol β complex. The incubation was at 37°C. Aliquots were withdrawn at 20 and 40 min in experiments without APE1, or 5 and 10 min with APE1, as indicated. The reaction products were analyzed as in Fig 1. The positions of the unligated BER product and ligated BER product are indicated. (TIF) [file pone.0124269.s004.tif]

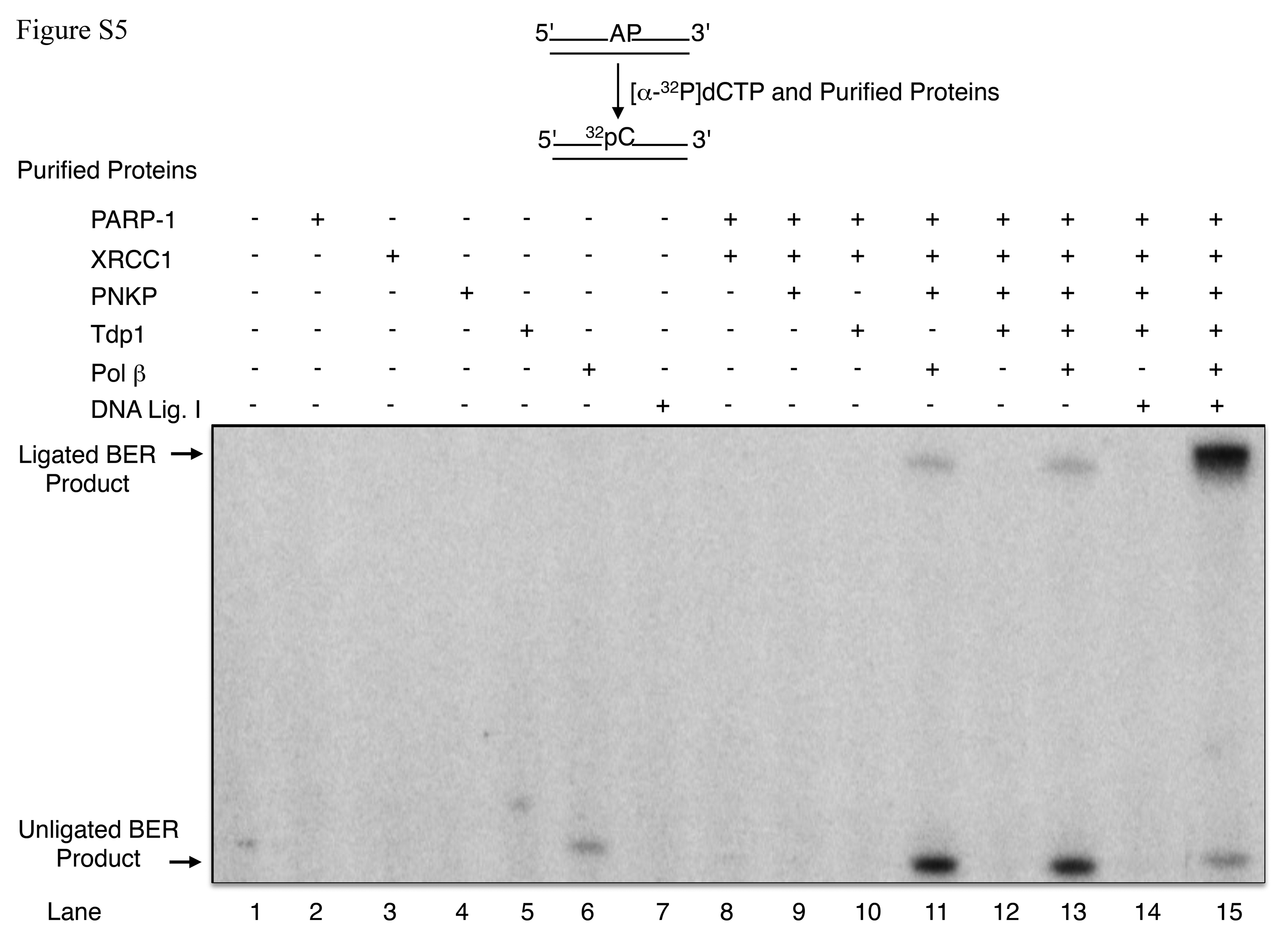

Supplement: S5 Fig — A schematic representation of the DNA substrate containing the AP-site and the reaction scheme is illustrated at the top. The reaction conditions and product analysis are described under Materials and Methods. The repair reaction was assembled on ice with (+) or without (-) purified BER factors alone or in various combinations, as indicated at the top of the phosphorimage. Repair was initiated by transferring the reaction mixtures to 37°C, and incubation was for 40 min. The reaction products were analyzed as in Fig 1. The positions of the unligated BER product and ligated BER product are indicated. (TIF) [file pone.0124269.s005.tif]

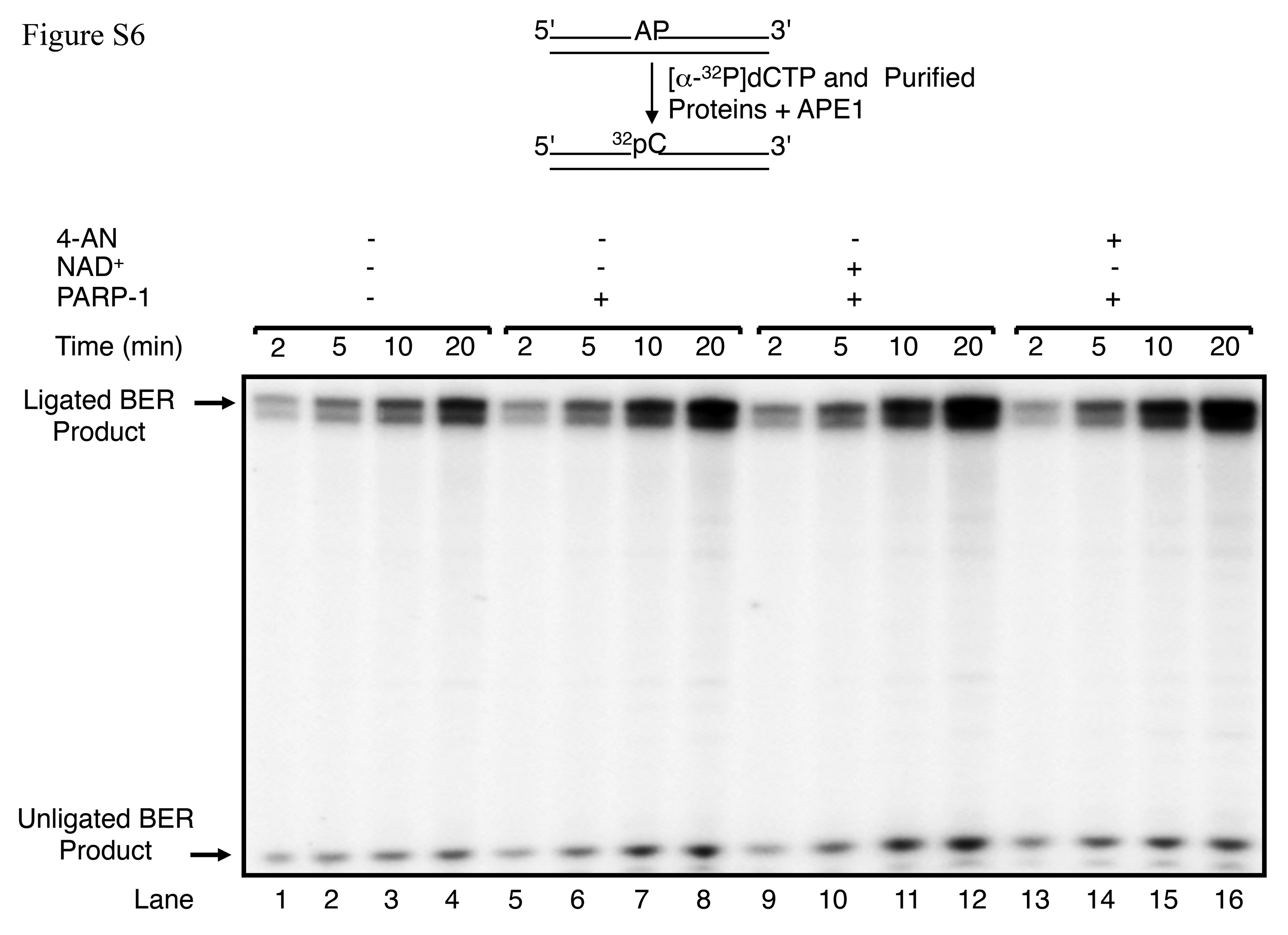

Supplement: S6 Fig — A schematic representation of the AP-site DNA substrate and the reaction scheme is illustrated at the top. The reaction conditions and product analysis are described under Materials and Methods. Repair reactions were supplemented either with (+) or without (-) 200 nM PARP-1, NAD+ (100 μM), or the PARP inhibitor 4-AN (100 μM), as indicated. The repair was initiated by transferring the reaction mixtures to 37°C. Aliquots were withdrawn at 2, 5, 10 and 20 min, as indicated. The reaction products were analyzed as in Fig 1. The positions of the unligated BER product and ligated BER product are indicated. In the reaction mixtures with the PARP-1 inhibitor 4-AN, PARylation was inhibited > 95%. (TIF) [file pone.0124269.s006.tif]

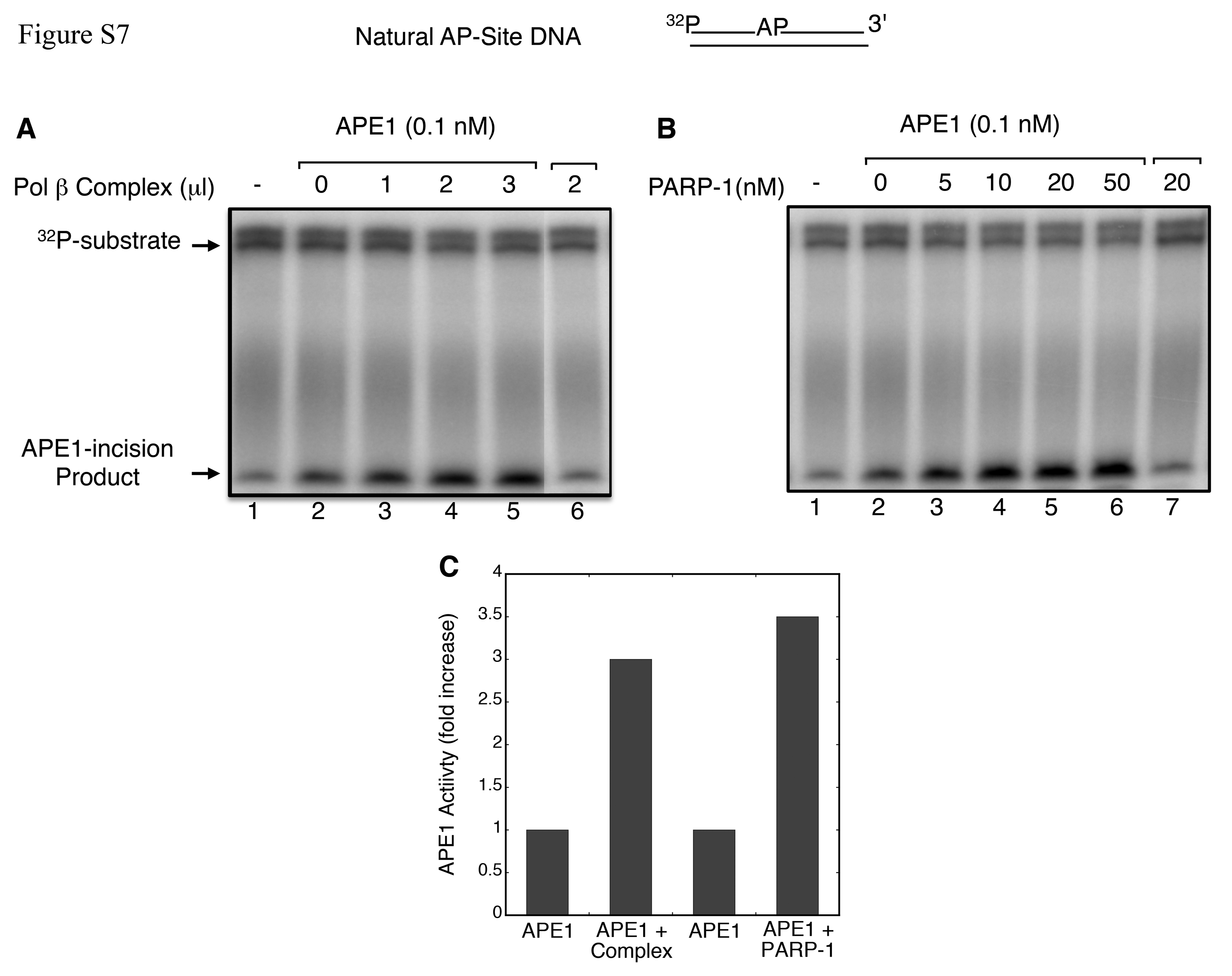

Supplement: S7 Fig — A schematic representation of the DNA substrate containing the natural AP site (AP) is shown at the top. The reaction conditions and product analysis are described under Materials and Methods. (A) APE1 incision reactions were assembled on ice either with increasing amounts of pol β complex (A) or with increasing amounts of purified PARP-1 (B). The incision reaction was initiated by addition of APE1 and transferring the reaction mixtures to 37°C for 10 min. The reaction products were analyzed as in Fig 1. The positions of the 32P-labeled substrate and product of APE1 strand incision are indicated. (C) Quantification of APE1 product formation at the highest amount of the pol β complex (3 μl) and the highest concentration of PARP-1 (50 nM) revealed an approximately 3-fold increase in APE1 activity as compared to that of APE1 alone. (TIF) [file pone.0124269.s007.tif]

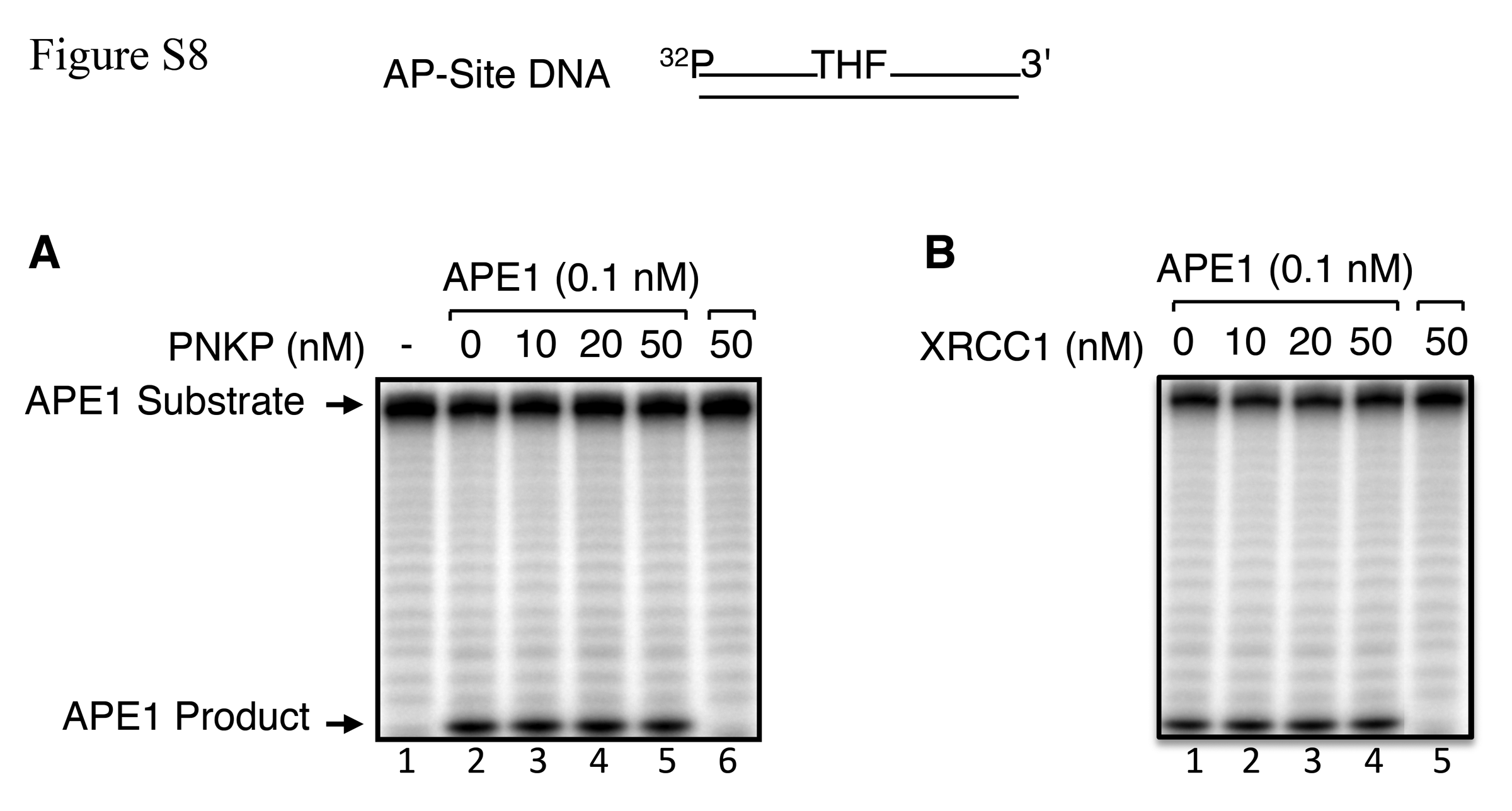

Supplement: S8 Fig — A schematic representation of the APE1 DNA substrate containing THF is illustrated at the top. The reaction conditions and product analysis are described under Materials and Methods. The APE1 incision reaction mixture was assembled on ice, either with increasing amounts PNKP (A), or XRCC1 (B), as indicated. The minus APE1 control lane is indicated. The incision reaction was initiated by adding APE1 and transferring the reaction mixtures to 37°C. After 10 min incubation, the reaction products were analyzed as in Fig 1. The positions of the 32P-labeled substrate and product of APE1 strand incision are indicated. Lane 1 in panel (A) represents substrate alone. The results of this analysis showed no increase in APE1 activity with addition of PNKP or XRCC1 as compared to that of APE1 alone. (TIF) [file pone.0124269.s008.tif]

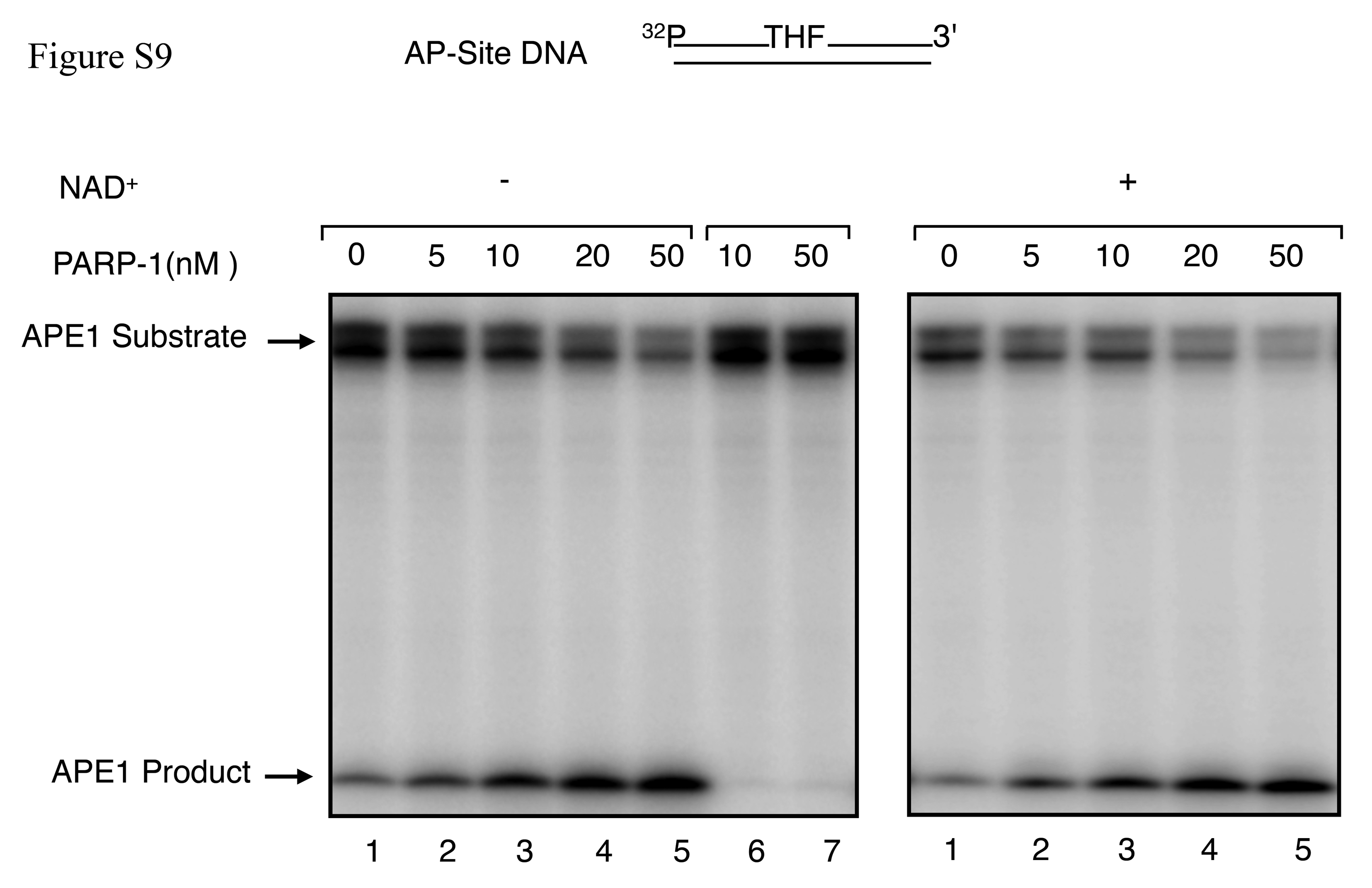

Supplement: S9 Fig — A schematic representation of the DNA substrate containing THF is illustrated at the top. The reaction conditions and product analysis were as described under Materials and Methods and S7 Fig. The incision reaction mixture (10 μl) was assembled on ice with 32P-labeled AP-site DNA (50 nM) and increasing amounts of PARP-1 with (+) or without (-) NAD+, as indicated. Lanes 6 and 7 are minus APE1 controls. The incision reaction was initiated by addition of APE1 to the final concentration of 0.1 nM and transferring the reaction mixtures to 37°C. After 10 min incubation, the reaction products were analyzed as in Fig 1. The positions of the 32P-labeled substrate and product of APE1 strand incision are indicated. Quantification (not shown) of the APE1 products demonstrated an approximately 3-fold increase in APE1 strand incision activity as compared to that of APE1 alone, whereas the activity was not influenced by NAD+. (TIF) [file pone.0124269.s009.tif]

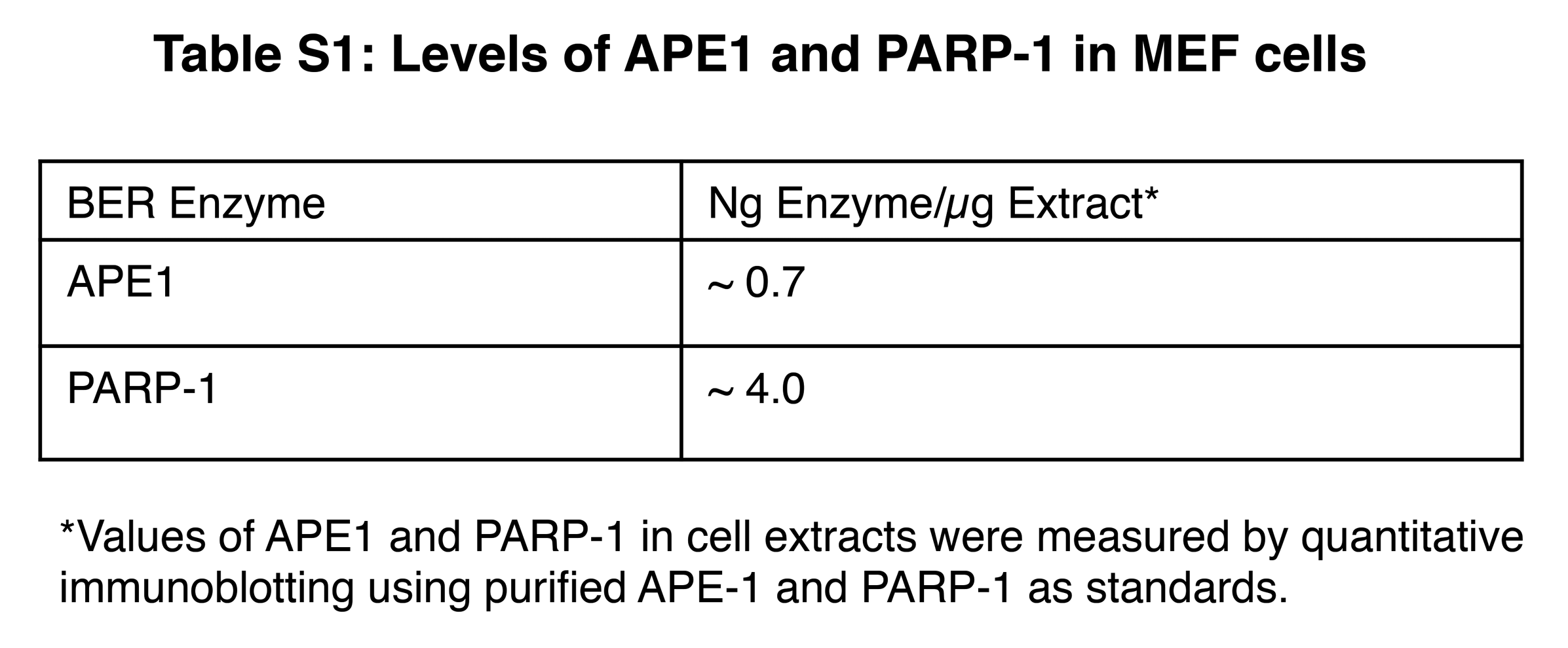

Supplement: S1 Table — Values of APE1 and PARP-1 in cell extracts were measured by quantitative immunoblotting as described under Materials and Methods using purified APE1 and PARP-1 as standards. (TIF) [file pone.0124269.s010.tif]
